# Embedding Public Involvement in a PhD Research Project With People Affected by Advanced Liver Disease

**DOI:** 10.1111/hex.14097

**Published:** 2024-06-12

**Authors:** Cathy J. Beresford, Mahabuba Rahman, Yvonne Gray, Sandra Ramshaw, Leslie Gelling, Sue Baron, Jackie Dominey

**Affiliations:** ^1^ Department of Nursing Science, Faculty of Health & Social Sciences Bournemouth University Bournemouth Dorset UK; ^2^ Member of the Public Involvement Group; ^3^ Lewis‐Manning Hospice Care Poole Dorset UK

**Keywords:** collaboration and coproduction, embedded consultation, liver disease, participatory research, PhD research, public involvement

## Abstract

**Background:**

Liver disease is an increasing cause of morbidity and mortality in the United Kingdom and can be challenging to live with in the advanced stages. There has been little research exploring the healthcare experiences of UK individuals with decompensated disease when the liver cannot carry out its functions properly. A PhD research project was developed with people who have liver disease to explore care experiences in decompensated advanced liver disease. Public involvement (PI) is an essential aspect of meaningful health research, and this paper reports on the progression of our PI approach in this ongoing study.

**Objective:**

To embed PI throughout the research project to ensure that the study is meaningful to individuals with liver disease and the people who support them.

**Methods:**

The research adopts a Constructivist Grounded Theory methodology to develop a theory of care experience. Various PI approaches were considered in developing the PI strategy for this qualitative study. Initially, *Embedded consultation* was the preferred model, which has evolved to include aspects of *collaboration and coproduction*. A PI group was set up to oversee the project through the national public engagement website VOICE, and reflections on PI from three members of the group are included in this paper to illuminate the PI process.

**Results:**

Six individuals with liver disease and three carers from across the United Kingdom are part of an ongoing PI group. Their role includes commenting on the findings of the systematic literature review for this project and contributing to decisions about recruitment, data collection and data analysis. Additionally, they had a direct impact on changing the focus of the research. The PI group will continue involvement until the completion of the project.

**Conclusion:**

Successfully embedding PI into doctoral research, as demonstrated in this project, requires commitment, planning and dedication to reciprocal working for the benefit of PI contributors as well as the research. This approach could be adopted by other postgraduate researchers.

**Patient or Public Contribution:**

This project is overseen by the PI group, whose contribution is described throughout, including reflections from three PI group members.

## 1 Introduction

The purpose of this paper is to report on the public involvement (PI) strategy of this ongoing PhD study with people who have primary liver disease of various aetiologies. By sharing insights from this project, the aim is to inspire fellow postgraduate researchers to effectively integrate PI strategies into their own work. The project is a collaboration between the Department of Nursing Science at Bournemouth University and Lewis‐Manning Hospice Care and is due for completion in autumn 2025. The aim of this research was to develop a comprehensive understanding of the care experiences and perspectives of individuals with decompensated advanced liver disease, their carers and the professionals involved. Through this exploration, the goal is to construct a theory of care that will serve as a framework for understanding the diverse needs of individuals navigating the complexities of advanced liver disease. This theory will further facilitate the development of tailored healthcare interventions aimed at improving the quality of care provided to this population.

In recent years, the importance of PI has been increasingly recognised as integral to health research and as a requirement for funding and research ethics approval. From the outset of the study, it was decided that one of the research objectives would be to embed PI so that the research would be meaningful to people experiencing liver disease and the professionals and carers who support them. Initially, the model of PI considered appropriate was *embedded consultation* [1] because the lead researcher had overall responsibility for decisions made for the PhD. However, as the project progressed, PI had become more fundamental to the research with principles of *collaboration and coproduction* [1] clearly apparent in the approach.

## 2 Background

### 2.1 Liver Disease

In the United Kingdom, liver disease is a major cause of illness and death [2], with an unpredictable disease trajectory and uncertainty as a major aspect of living with advanced liver disease [3, 4]. The main types are alcohol‐related liver disease, metabolic dysfunction‐associated steatotic liver disease (MASLD) and infection with hepatitis B or C viruses [2]. In the advanced stages, liver disease is characterised by widespread scarring and damage, which inhibits normal function. Sometimes referred to as cirrhosis of the liver, the disease can be compensated (whereby the liver is scarred but can still function reasonably) or decompensated. In decompensated liver disease, the liver cannot undertake its functions properly [5]. At this stage, people may experience numerous symptoms including pain, breathlessness, muscle cramps, sleep disturbance, depression, anxiety and sexual dysfunction, as well as social and financial issues [6, 7]. These symptoms combine to impact an individual's quality of life and need for support.

Access to palliative (supportive) and end‐of‐life care for people with advanced liver disease is variable, with inequalities apparent [8]. There has been little focus on the experiences of care received by people with advanced liver disease and it has been recommended that more research is needed to explore the perspectives of people receiving care [9]. A recently published literature review conducted as part of this study found variations in the care experiences of people in the United Kingdom with advanced liver disease towards the end of life, an overall lack of access to specialist palliative care services and lack of clarity about when the care of people with liver disease becomes palliative or end of life [10]. Conversely, when services for people with liver disease were reported to be person‐centred, people's experiences were found to be more empowering. These findings, and discussions with a PI group (set up to oversee the project), the PhD supervisory team and hepatology healthcare professionals, led to a change in the focus of the study to care experiences in decompensated advanced liver disease. Having received Research Ethics Committee (REC) approval, the PhD project is currently in its second year, in the recruitment and data collection phase.

The study objectives are as follows:

1. To explore the subjective experiences and perspectives of:
  a. Individuals with decompensated advanced liver disease receiving care.
  b. Carers (such as partners or other family members) of people with decompensated advanced liver disease.
  c. A range of professionals providing care to people with decompensated advanced liver disease.
2. To embed PI throughout the research project.

Objective 2 is the focus of discussion in this paper.

### 2.2 Public Involvement

Sometimes described as patient and public involvement (PPI), the decision to include PI as one of the research objectives arose from a commitment to ensuring that the project is meaningful to individuals affected by liver disease. According to the National Institute for Health Research (NIHR) [11], PI means research being carried out ‘with’ or ‘by’ members of the public, rather than ‘to’, ‘about’ or ‘for’ them. Research studies that effectively involve the public have been described by the Health Research Authority [12] as:

- Being more relevant to the people participating in the study.
- Being designed in a way which is acceptable to the people participating in the study.
- Having participant information which is understandable to those taking part.

In defining PI for this paper, we have adopted the NIHR's [11] use of the term ‘public’ to include *‘*patients, potential patients, carers and people who use health and social care services as well as people from specific communities and from organisations that represent people who use services. Also included are people with lived experience of one or more health conditions, whether they are current patients or not.’

It is ethical to include PI in research as part of respecting the rights, safety, dignity and wellbeing of individuals participating in the study, which is expected by RECs [12]. Other PhD researchers have demonstrated dedication to effectively implementing PI into their research to enhance the quality and relevance of the doctoral research [13, 14]. In liver disease, Hull et al. [15] describe the increasingly important role of PI in the development and implementation of research, yet PI was sometimes found to be missing from studies in the systematic review undertaken as part of this project [10], reflecting Ocloo et al.'s [16] argument that there are still considerable barriers to PI that need to be addressed.

In undertaking this project with people who have advanced liver disease, it was deemed essential to use an empathetic and inclusive approach in giving people a say in how they experience their care. This made us determined to be thoughtful and sensitive and to work in a participatory way with the different stakeholders. The PI strategy has become fundamental to the philosophical approach of the project. This is a qualitative research study that explores people's experiences and perspectives of the care they receive when they have advanced liver disease. Principally, this research values knowledge constructed from lived experience [17, 18]. Part of this knowledge construction is through interviewing people about their experiences of care using Constructivist Grounded Theory, which recognises the subjective nature of reality and the importance of individuals' perspectives ([19], but PI in *how* the research is designed and conducted has become a crucial component of the methodology. This project is about coconstructing knowledge to develop theory both through PI and through data collection—it is all connected, guided by a philosophical approach that underpins the research.

### 2.3 Lived Experience

In his theory of knowledge, distance and experience, Beresford [17] proposes that knowledge is more likely to be reliable or accurate the smaller the distance between direct experience and interpretation. The lead researcher does not have liver disease, is not a carer for someone with liver disease and does not have a background in hepatology. Thus, they have positioned themselves as an outsider acknowledging that they do not know what individuals with liver disease are going through, and respecting people who have real‐life or lived experiences of this as the experts. This recognition of distance from the lived experience of liver disease has driven the desire to seek and listen to the views of people with liver disease, and the people who support them, in developing the project. Lack of experience of the lived situation has an impact on understanding and interpretation of the experience [17]. However, Beresford [17] suggests that some factors better equip people to get closer to the experience of others, even when they do not have it themselves. For example, if they have experienced another form of oppression or discrimination. The researcher considers that her life experiences—as a nurse, a researcher, a mother and an individual with experience of other health conditions—do provide skills in understanding other people's experiences more effectively. Furthermore, Beresford [17] proposes a set of value‐based principles, such as listening and being empathetic, which underpin this project.

## 3 Methods

### 3.1 Deciding on a PI Strategy

In the early stages of this liver disease project, the lead researcher undertook the module *Public Involvement in Research*, which is coproduced by members of the Public Involvement in Education and Research (PIER) Partnership at Bournemouth University. This was invaluable in shaping both the approach to the project and the PI strategy. The module was cofacilitated by people with lived experience of different health conditions, and students were encouraged to put themselves into the shoes of the people they were doing research with. Students were required to seek individuals with lived experience to discuss their research and to consider their PI strategy. Initially, this was challenging, and a range of strategies were implemented, including social media, meeting healthcare professionals in the field and shadowing a hepatology specialist nurse in the clinic to meet individuals with liver disease and hear their perspectives.

It has been argued that specific definitions and approaches to PI in research are frequently tokenistic, unclear and secondary to the central research process [1]. This was a motivating factor in the decision to include PI as one of the research objectives for the study. The addition of this objective has encouraged the research team to be continually reminded of this core component of the project, providing strength and justification for PI throughout.

In deciding which PI approach to use, the first step was to properly understand what constitutes PI. A good starting point was the NIHR [11], which describes it as ‘research being carried out with or by members of the public, rather than to about or for them’. Understanding this changed the way we spoke and wrote about the project. Instead of describing it as *about* people's experiences of care in advanced liver disease, there was a shift to describing the research as being undertaken *with* people who have liver disease. This small change is important because it is increasingly recognised that language matters in health and social care in terms of how professionals communicate with individuals to reduce stigma and enhance effective care [20]. Liver disease is a stigmatised condition [21], particularly as it is primarily caused by alcohol use, being overweight or viral hepatitis [22]. The team acknowledges the importance of working with people and demonstrating appreciation for their value. By changing the way the research is described, we ensure that this principle remains central to our research approach.

In their concept analysis of PI in health and social sciences research, Hughes and Duffy [1] developed five operational definitions of PI, which were explored in developing the PI strategy for this project:

1. Undefined involvement
2. Targeted consultation
3. Embedded consultation
4. Collaboration and coproduction
5. User‐led research

From the outset, it was clear that *undefined involvement* was not appropriate because this is when research is done *to* people rather than *with* them [1], which conflicted with our research objectives. *Targeted consultation* involves limited involvement with individuals receiving little or no updates following their PI input [1]. This model seemed too superficial and unlikely to be mutually beneficial to the people taking part in PI or, therefore, to the quality of the research being undertaken. We were keen to involve people throughout the research process with a more reciprocal arrangement to suit both the researchers and the individual members of the PI group; therefore, this approach was too restrictive.

*Embedded consultation* is a PI approach where individuals with lived experience are regularly consulted throughout the research process, with the involvement strengthened when several people with a range of views are included. In this model, the research team still has ownership and control over the study but actively participates in meaningful discussions with others [1]. In the early stages of this project, we decided that *embedded consultation* would be the most appropriate PI strategy because this is a PhD project for which the lead researcher is being funded, with the reality that she and the supervisory team have overall ownership and control. This is in contrast with *user‐led research*, which could have been appropriate if someone with lived experience of liver disease had led the research.

*Collaboration and coproduction*—In this model of PI, people with relevant lived experience are more equally involved as members of the research team [1]. This includes contributing to key decisions throughout the research process and receiving appropriate training to be able to do so. The researchers initially felt that because funding was received to carry out this PhD research, overseen by the supervisory team, the *collaboration and coproduction* model was not feasible. It is necessary to be pragmatic, transparent and honest about the approach to the research, so it was considered unrealistic for the study to be collaborative and coproduced. However, as the project has evolved, it has been discovered that it is often possible to work in a collaborative and coproduced way even if this is not the dominant model being applied. Hughes and Duffy [1] advocate that researchers should be open to relinquishing and sharing control to facilitate new ways of working. We are open to this because although it is necessary to retain ultimate control over the project, there are still ways of facilitating others to have influence at various stages of the research process. Collaboratively authoring this paper serves as an example of this approach.

### 3.2 Setting Up a Public Involvement Group

Once it was decided that *embedded consultation* was the appropriate model of PI, the PIER officer at Bournemouth University supported the research team to set up an online PI workshop via the national public engagement website VOICE to reach a range of individuals (Appendix S1). Eight people from across the United Kingdom responded to the invitation—five individuals with liver disease and three carers of individuals with liver disease. None of these individuals knew each other before the meeting. A 90‐minute workshop was held to discuss the findings of the systematic review [10] and to consider the development of this research.

The importance of treating people with respect when you involve them in PI has been emphasised by other researchers [23]; the research team were very aware of this when preparing for the VOICE meeting. In a previous role, the lead researcher had over 10 years of experience in facilitating a diabetes patients' advisory group. Those meetings were held in person and were very sociable, with refreshments provided and more opportunities for informal chat so that people got to know each other. It was unclear if this could be replicated through an online meeting.

In line with the guidance about PI [1, 13], each person who took part in the meeting was provided with a £25 voucher for their time, and this is the case for subsequent PI meetings, using the PhD research budget [24]. At the initial meeting, the first 20–30 min were spent on introductions, to give people the opportunity to share their background, why they wanted to be involved and to enable them to feel comfortable in the group. Having clear definitions and understanding of roles has been found to be important in PI [16], so the purpose of the meeting was clearly explained and a PowerPoint presentation about the project was delivered before further group discussion. Afterwards, everyone who attended the workshop said they would like to contribute further and take part in future meetings.

The identification of clear goals to clarify the purpose of PI in the research has been advocated [1], and our goal is to make the study meaningful to people with liver disease, and the people who support them. Opportunities for involvement include participation in the PI meetings, but also through email liaison where members are kept up to date with the progress of the project and asked for their opinions and suggestions. The role of the group so far has included making decisions about participant inclusion criteria, providing ideas for recruitment strategies and supporting the development of the interview schedule and participant information leaflets. They have had some involvement in early data analysis and will support with dissemination of the findings as the research progresses.

Using Rolfe, Freshwater and Jasper's [25] reflective model, Table 1 demonstrates the impact that PI is having on this project. Writing this journal paper encouraged the research team to think creatively about ways of involving people, including the creation of a PI newsletter. The first issue was sent to the group in January 2024 (Appendix S2). The group has agreed that the newsletter will be six monthly to include updates on the project, and members will have the opportunity to contribute.

**Table 1:** Public involvement impact using Rolfe, Freshwater and Jasper's [25] reflective model.

When	What?	So what?	Now what?
October–November 2022	Undertook the Public Involvement in Research module at Bournemouth University.	This unit increased the understanding of the theoretical and practical aspects of public involvement in research. We explored why it is important and how to go about it. Met other postgraduate researchers and the unit was cofacilitated by people with lived experience of different health conditions. This was an opportunity to share the research with the group and work out ways of incorporating public involvement into the project in a meaningful way.	Public involvement will be embedded into this PhD research project and included as one of the research objectives.
			Next steps: 1. Speak with a range of different stakeholders regarding the project to gain a variety of perspectives. 2. Seek opportunities to meet people with lived experience of liver disease to ensure the project is meaningful to them. 3. Consider the language used in the project to ensure it is person‐first with an emphasis on working *with* people.
January 2023	Shadowed a hepatology nurse specialist in her clinic (two occasions).	Met six different individuals with liver disease (of different aetiologies). One of them has advanced liver disease. Gained insight into positive and negative aspects of care; for example, relationships with healthcare professionals and access to information. Challenges include regional variations in access to services. Explained the project to some of the patients, who said it sounded interesting and useful.	This provided confidence that the project was worthwhile. The conversations with both patients and healthcare professionals at the clinic provided insight into some of the challenges people face living with liver disease, how people's needs can be met and where there may be gaps in services.
			Next steps: Continue systematic literature review and seek formal public involvement.
January 2023	In the clinic (as a Diabetes Specialist Nurse), consulted with a man with liver disease and his daughter.	In this consultation, the focus of care was supporting this gentleman to manage his type 2 diabetes. Mentioned the liver disease project and they described some of the challenges they have faced in accessing services for his liver disease. Wrote to the GP to arrange a referral to the hepatology clinic. It was helpful to hear their perspectives and consider some of the challenges they face—for example, the hepatology clinic is over a 30‐minute drive, and the gentleman is not well enough to drive there himself.	Although this was not formal public involvement in the project, it was useful to speak with this gentleman and his daughter about their experiences because it encouraged reflection on some of the issues important to them, such as how they access services, and that some aspects of care seem disjointed.
			Next steps: Arrange a formal public involvement opportunity.
February 2023	Submitted an opportunity for public involvement to VOICE (https://voice-global.org).	Submitted a request for people with liver disease, or experience of supporting someone with liver disease, to be involved in a focus group/one‐to‐one discussion as part of developing the project.	Next steps: Liaise with the Public Involvement in Education and Research (PIER) officer for support with the PI opportunity.
			Gaining confidence about undertaking public involvement—it was helpful to have support from the PIER officer.
April 2023	Conducted the first online public involvement meeting (through VOICE).	Attended by four people with liver disease and four carers of individuals with liver disease. Prepared carefully for the meeting at which we all introduced ourselves and focussed on two aspects: 1. The systematic literature review. 2. How the research project should move forward. All of the group agreed to be involved further as part of an ongoing public involvement group.	1. The feedback on the systematic literature review was helpful and is discussed in the published paper. 2. We agreed: a. Who should be interviewed for the research (patients, carers and HCPs). b. What sort of questions they should be asked. 3. The meeting was extremely useful in shaping ideas and thinking about what is important to people with liver disease and their carers. 4. It was apparent from the discussion that there is a lack of clarity about palliative and end‐of‐life care and that it will be difficult to focus on these aspects specifically. This was apparent from the systematic review and discussions with HCPs as well. Next steps: Change the focus of the research. Write protocol and apply for ethics approval with support from the group.
			The next PI meeting is in November 2023.
June 2023	Oral and poster presentation at the Faculty of Health and Social Sciences Postgraduate Research conference.	Presented the public involvement strategy to other postgraduate researchers and academics at the university.	This was helpful to shaping ideas more clearly, and sharing the work with others so that they might also incorporate public involvement into their research. This experience increased confidence and it was good to get feedback about it from peers.
			Next steps: Seek other opportunities to share the PI approach.
June–July 2023	Email liaison with the public involvement group as part of ethics application and participant information leaflets.	Four members of the PI group offered feedback on the participant information leaflet, such as: The need to make it clear that individuals are required to communicate with me in English for interview. Discussion around the title of the research and how to make it clear. A grammatical error in the leaflet. Changing certain words, for example, replace the word ‘tummy’ with the word ‘abdomen’ in relation to ascites. One member said: ‘the participant leaflet is clear and understandable.’	Changes were made to the participant information leaflet as suggested by members, or if there was a justification for not making the change this was explained. For example, the difference between the short title and the long title was explained, and the reason that the word ‘decompensated’ is not used in the short title is that it may not be understood by everyone with liver disease.
			Next step: Let the PI group know when ethics approval is obtained.
July 2023	Emailed the PI group.	Update on the progress of the ethics application sent to the group to keep them in the loop.	Next step: Update the group when more information is available.
October 2023	Emailed the PI group on two occasions.	Informed the group about the: 1. Approval from the NHS Research Ethics Committee. 2. The successful publication of the systematic literature review and thanked them for their involvement.	Next step: PI meeting in November 2023.
October 2023	Public engagement: Presented to PIER members at the Having a Voice in Research course (Bournemouth University).	Presented the public involvement strategy to individuals with lived experience of different health conditions and answered their questions.	It was great to meet people with different experiences and to see how they received the research. A couple of people expressed possible challenges in recruiting people to the study because individuals with advanced liver disease can be very unwell—this is something to further consider and to think carefully about how people can be reached.
November 2023	Public engagement: Presented the project to the LIVErNORTH support group at their online meeting.	Fourteen people attended and the meeting was recorded for sharing. Received positive feedback—people said they found it interesting and asked questions.	Next steps: Come back once the results of the study are available to share the findings with the group. LIVErNORTH have agreed to share the recruitment advert in their newsletter and via social media.
November 2023	Second public involvement group meeting held online.	Five people attended today (three apologies). Presented a PowerPoint with an update on the project, and we reflected on the last meeting. Issues discussed included stigma and honesty about liver disease from healthcare professionals. We discussed recruitment. The group asked what support the researcher receives because some of the interview content can be quite emotional.	1. The discussion today influenced the researcher's thinking around issues of stigma, communication and honesty. It is interesting to note that some of the issues emerging in the PI meetings are the same as those emerging from the research data. 2. As suggested by the group, seek further opportunities to recruit people through a range of strategies. 3. Reflect on the researcher's self‐care whilst conducting the research—it was helpful that the group raised this issue and encouraged taking breaks and reach out for support.
			Next steps: Email the group with an update in January. The next PI meeting March 2024.
January 2024	Contacted by social media by a gentleman interested in participating in the research.	He does not meet the inclusion criteria (to participate in the research) because his liver disease has not progressed to decompensated. Mentioned the public involvement group and he said he would like to be involved. We had an email discussion about liver disease services and where people go for support.	This gentleman has shared the research advert with other people who may be interested in taking part. Some of the issues he raised about the care people receive for liver disease are similar to those emerging from the research data.
			Next steps: Invited him to attend the next PI meeting in March. Recruited an individual to take part in the research following recommendation from PI.
January 2024	Created a public involvement newsletter to share with the group and update them on the project.	This could be a positive way of sharing information with the PI group, and also demonstrating that their input is valued.	Next steps: At the March PI group meeting, ask the group how often they would like to receive the newsletter, what they would like it to include and whether they would like to contribute anything themselves.
January 2024	Contacted members of the PI group who attended both meetings to see if they would like to contribute to the journal paper for Health Expectations.	Have suggested that a couple of members might like to contribute *who you are and why you wanted to be involved in the public involvement group, what you feel the groups contribution is and anything you have got out of the experience so far. There might be other things you want to say*.	Three members of the group have provided contributions to the paper.
			Next steps: PI meeting scheduled in March 2024.
March 2024	Third public involvement meeting held online.	Five members of the group attended the meeting today. Explored early insights from the interviews conducted so far. Discussed empowering and disempowering experiences in liver disease care, as this is emerging from the data.	1. Some initial themes emerging from the data correspond with the experiences and perspectives of the group. 2. However, it is early days and important to avoid premature conceptualisation. 3. Issues around late diagnosis, inconsistencies in access to information and variations in care are apparent both in the data and amongst the members of the group. Next steps:
			Continue data collection.
			PI Newsletter every 6 months for the duration of the project.
			PI meeting scheduled for June 2024.
March 2024	Liaison with group members about avenues for recruitment.	One member of the group provided suggestions for organisations to approach regarding recruitment.	Emails sent to suggested organisations.
			Next steps:
			Continue seeking opportunities for recruitment to the study.
April 2024	Presented *Public involvement in postgraduate research* at the Department of Nursing Science Research & Scholarship seminar.	Lunchtime presentation to other postgraduate researchers to explain the public involvement strategy for this project. Shared insights from PI group members.	Next steps:
			Suggestions were provided for recruitment opportunities for the study. Another person was recruited to take part in the research following a recommendation from the PI.
April 2024	Health Expectations submission—received peer review. Liaison with PI contributors via Teams and email.	Peer review feedback from the journal paper was carefully considered and revisions made.	Next steps:
			Submit the revised manuscript by the beginning of May 2024.
			Next PI meeting June 2024.
			Present PI strategy at the Faculty of Health and Social Sciences postgraduate researcher conference.

## 4 Results

PI is an important aspect of this project, as illustrated in Table 1. The PI group is made up of individuals from across the United Kingdom who did not know each other prior to joining. Table 2 illustrates the characteristics of the group's members. All members have access to the internet and can use Microsoft Teams to participate in PI meetings. Eight people were invited through VOICE and one person was subsequently invited through Facebook.

**Table 2:** Characteristics of PI group members.

Gender	Ethnicity	Age (at the first PI meeting)	Connection to liver disease
Female	Asian British	39	Has liver disease
Female	Asian British	41	Has liver disease
Male	White British	73	Has liver disease
Female	White British	68	Has liver disease
Female	White British	73	Has liver disease (and has been a carer for someone with liver disease)
Female	Asian British	42	Carer for person with liver disease
Female	White British	69	Carer for person with liver disease
Female	White British	Not provided	Carer for person with liver disease
Male	White Welsh	Not provided	Has liver disease

Although the focus of the research is decompensated advanced liver disease, the research team decided it would be appropriate for the PI group to include individuals with liver disease at an earlier stage because the disease trajectory is unpredictable, and because individuals in the decompensated stage might not be well enough to take part in PI [3]. Carers were invited to take part because their unique insights are valuable [26], and they may have experience supporting an individual who has died of liver disease, or who is too unwell to participate in PI themselves. Three members of the PI group have provided their reflections on taking part in the project to date.

## 5 PI Group Members' Reflections on the Liver Disease Project

### 5.1 PI Group Member Mahabuba Rahman

I am a 39‐year‐old Bangladeshi woman living in Tower Hamlets, London, blessed with the joys of being a mother to three wonderful daughters. My health conditions have shaped my perspective on life and given me the desire to learn more about myself, my condition and others around me.

Being involved in the liver disease project is a personal choice for me to actively contribute to community issues as well as share my life experiences, bring in cultural diversity and gain a sense of empathy for others on a similar journey. When I saw the advert on the VOICE website, I wanted to take part in the workshop because of my own experience of liver disease.

I have multiple health conditions, and I find that it can be hard to get information about liver disease. English is not my first language, but I can understand and speak it well and there is just not enough information out there. I thought that taking part in the project would be a source of support and learning. It is also helpful to receive the £25 voucher for each meeting.

Taking part is different from attending a health education class where you are told what to do; I can gain information from others, share my experiences of liver disease and influence the research. In this group, I contribute my insights, which may resonate with others facing similar health challenges. I believe that sharing my story is powerful and extremely supportive of our community, and we can learn from each other. The group is not only a platform for discussion and contribution to the research project but also a place for knowledge to be exchanged. We can raise awareness, share our perspectives, help shape the liver project and benefit from the advice we receive from others.

My health has become a unique part of me. Reflecting on my journey in this project so far, I have been able to comfortably discuss my health journey and life experiences with people facing similar battles. This is invaluable to me, and it is very positive to see how the voices of individuals come together. I am not just a Bangladeshi woman in Tower Hamlets facing health challenges; I am an advocate for change, driven by a sense of purpose and shared responsibility for the wellbeing of our society.

The meetings are an informed space for individuals to come and talk about the complexities of liver disease without fear of being judged. By speaking with members of this group, the researcher can better understand the needs of people with liver disease to shape the study. It is about bringing all our views and knowledge together to make things better for people with liver disease and the wider community. This research is Cathy Beresford's PhD, but it is our shared project.

### 5.2 PI Group Member Yvonne Gray

I am a 68‐year‐old retired primary school headteacher from Sunderland, diagnosed with the liver disease metabolic dysfunction‐associated steatohepatitis (MASLD/MASH) in 2010. The disease was previously known as NAFLD/NASH. I have also been a carer all my life for family members with other health conditions.

When I was diagnosed, little was known about MASLD, let alone any research being done on it. It has only been through my diagnosis and involvement as a governor of the LIVErNORTH Charity that I have learned about liver disease in general. Although common, MASLD is a largely silent disease that sneaks up on patients. Apart from fatigue and constant discomfort in the upper right side of my abdomen, I was only aware of symptoms of my other conditions, not realising that my, so far undiagnosed, liver disease was progressing. I had ‘mild changes’ in my liver function blood tests and was only referred to a liver specialist after another consultant, dealing with my other conditions, asked for their opinion. Following a biopsy, I was shocked to be told my liver was at stage three of a four‐stage disease—the fourth stage being cirrhosis.

The reason I was keen to be part of the liver disease project is perhaps for obvious, personal reasons: I am fearful of the ‘unknown’ as my own, fairly advanced, liver disease progresses to a decompensated cirrhosis stage. I felt that my personal experience as a patient, and a lifelong carer, would help other members of this group, and the research, directly and indirectly.

I am acutely aware that research offers hope of better diagnostic tests so people are identified sooner, and the possible discovery of new treatments could prevent advanced liver disease, or even reverse it. Moreover, research offers the chance to affect change whether in diagnosis, in care or in reducing stigma. We all have a voice and a choice—these enable us to work together for the benefit of all. One of the key issues emerging from this project, which deeply concerns and frustrates me, is the evident lack of consistency in the care individuals receive at different stages of their liver disease. Additionally, the experience of stigma significantly impacts not only their own but also their loved ones' physical and mental well‐being.

As an active member of LIVErNORTH, with a very close relative who has also recently been diagnosed with MASLD/MASH, I am passionate about involving more patients to get involved in research to drive meaningful change and reduce the stigma surrounding liver diseases. Having participated in PI previously, I found that some researchers seem to view it as a formality, and I have walked away from those groups questioning their impact. However, this project stands out due to the leadership of the researcher; her organisation and time management are outstanding. Importantly, she ensures that everyone has a chance to contribute, providing an environment where my voice feels valued.

### 5.3 PI Group Member—Prefers to Remain Anonymous

I am a 73‐year‐old retired civil servant from the North‐East of England with two daughters and a grandson. I have a range of health conditions, including liver disease, and my husband, who died in 2017, had alcohol‐related liver disease. I wanted to take part in this PI project due to my personal connection with the issues being addressed. My husband's challenges with alcohol and his mental health had a profound effect on me, raising questions and concerns within me about my role and responsibility in his struggles.

I am interested in understanding other people's experiences and perspectives, particularly if they share similar feelings of guilt or self‐blame. By contributing to the project, I gain insights into others' attitudes and experiences and also offer support and assistance where possible. So far, I have found the project interesting, and I appreciate the opportunity to share ideas about the condition and gain more understanding of the problems that liver disease can cause. Sometimes, people do not want to talk about liver disease because they fear they will be judged by others, as if they are to blame for their condition. Within this group, we have the opportunity to speak without judgement.

During the meetings, I have enjoyed interacting and talking to other people, which is important. It keeps me mentally stimulated to take part in activities like this, especially since I retired. I think it is important to reach out to people; communication is vital to my wellbeing. Furthermore, our perspectives and experiences deepen the researcher's understanding and empathy towards individuals grappling with liver disease, including those who are contending with alcohol‐related issues. We highlight the specific challenges that people face and provide ideas that help shape the research, for example, emphasising the importance of involving carers in the study.

I have contributed to PI in other research projects and have found that sometimes the online meetings are not well‐structured and can easily go off‐track. There might be one or two people who take over the discussion or move away from the specific issue we are supposed to be addressing. That has not happened in this group—meetings have been good, well‐structured and on point, which is the whole crux of a successful meeting.

## 6 Discussion

It is apparent from the reflections of the PI members that the motivation behind involvement in the liver disease project may differ between individual contributors and the research team but with clear overlap. The research team's main motivation for PI from the outset was to make the study meaningful and relevant to individuals with liver disease and the people involved in their care. More broadly, it was important to build relationships and give voice to people as part of coproducing knowledge. The lead researcher wanted to learn from the insights of the PI contributors, and the contributors themselves described a desire to provide those insights, as well as to learn from others, raise awareness of prominent issues and contribute to their own wellbeing and that of society. This is similar to the findings of Reynolds and Beresford [27], who found that PI is a social practice with diverse meaning and value that extends beyond health research. Knowles et al. [28] emphasised the importance of two‐way learning between the researcher and PI members as a mechanism for authentic coproduction. This is apparent in this project, which is not about the research team simply extracting information from the PI group, it is about developing trusting, reciprocal relationships and sharing knowledge for the purpose of coproduction.

Knowles et al. [28] encourage researchers to be willing to change the primary outcome of the research based on PI. This idea facilitated a shift in the study's focus after the systematic review, away from care experiences of palliative and end‐of‐life care specifically. It was clear from the discussion with the PI group that these terms can be ambiguous, especially because liver disease has an uncertain disease trajectory. Separately, the importance of including individuals with advanced liver disease, their carers and the professionals involved in their care as participants in the research project, was strengthened by the perspectives of PI members. In particular, the importance of the unique insights of carers was clear from the group. These examples of how PI is shaping the project demonstrate its importance to the ongoing research process rather than as an add‐on feature [13].

Other researchers have demonstrated the value of PI as an integral part of working collaboratively with people for the purpose of coproduction and to enhance the quality of the research. In the Born in Bradford research programme, Rahman et al. [29] argue that ‘nothing about us, without us: is for us.’ Like Dawson et al. [13], they discuss the importance of people being listened to, their experience and knowledge being valued and their input acted upon, which is illustrated in this project with people who have liver disease. Furthermore, when people feel heard and valued, they are more likely to engage further in the PI process [29], a phenomenon observed in this study. Following the initial PI workshop, individuals expressed their willingness to continue their involvement.

### 6.1 Challenges

In the early days of the project, we had to learn more about PI and how to make it work. It was extremely helpful having access to the *Public Involvement in Research* module at Bournemouth University to support learning and developing the PI strategy. However, such education may not be available to all researchers. Whilst other sources of information and support regarding research PI can be accessed [11], the lead researcher found the taught unit invaluable in developing understanding, increasing confidence and in engaging with people with lived experience.

Initially, it was challenging to reach people for the purpose of PI, and the use of the VOICE website proved vital. It is positive that the PI group is made up of various individuals from across the United Kingdom because we were keen to give a diverse range of people the opportunity to contribute and wanted to be inclusive to marginalised and seldom‐heard groups [16, 23]. However, we recognise that this PI group is less representative than we would have liked; for example, mostly women are involved even though men are more likely to have liver disease [30]. It has been argued by Ocloo et al. [16] that addressing equality and diversity is an overlooked aspect of PI, which needs to be more widely explored. This does not just apply to PI though, but is also apparent in research more generally. For example, liver disease is more common in certain ethnic groups [31], but in the systemised review [10], it was found to be mostly white people who participated in the studies. The reflections presented in this paper demonstrate that a strength of the PI in this research is the inclusion of individuals from diverse backgrounds and life experiences.

PI in this project so far has required careful planning and ongoing consideration of how best to integrate it into the research process. It takes time, and we sometimes worry about doing it ‘right’ because of anxiety to avoid misjudging situations, being tokenistic [23] or there being a mismatch between the priorities of the research team and the PI group. However, Knowles et al. [28] point out that for involvement to be authentic, there does have to be space for tension and disagreement at times. It can be complicated to coordinate meetings and work out how to involve people. For similar reasons, Rahman et al. [29] highlight that academics and researchers are sometimes resistant to coproductive PI. However, it has been helpful to have PI as one of the research objectives to encourage the whole research team to appreciate its value. Furthermore, because planning for PI started early in the project, it is difficult to imagine *not* including PI—it is a fundamental aspect.

## 7 Conclusion

PI is essential to ensuring that health research is meaningful, acceptable and understandable to people affected by it. Health researchers have a responsibility to effectively incorporate PI into their work, and this ongoing project demonstrates that PI can be interwoven into PhD research practice. Using *embedded consultation*, the research team have found ways to develop the PI strategy to include aspects of *collaboration and coproduction*, and our approach could be adopted by other researchers. From the perspective of group members, PI is an opportunity to gain knowledge, share experiences and build connections with other people, enhance personal wellbeing and contribute to society. The PI in this project continues to evolve and will be further reported on completion of the study.

## Author Contributions

**Cathy J. Beresford:** conceptualization, methodology, writing–original draft, writing–review and editing, investigation. **Mahabuba Rahman:** writing–original draft. **Yvonne Gray:** writing–original draft. **Sandra Ramshaw:** writing–original draft. **Leslie Gelling:** writing–review and editing, supervision. **Sue Baron:** writing–review and editing, supervision. **Jackie Dominey:** writing–review and editing, supervision.

## Conflicts of Interest

Thank you to the members of the public involvement group who are contributing to this PhD project, and to the Public Involvement in Education and Research (PIER) Partnership at Bournemouth University for their guidance and support.

## Supporting information

Supporting information.

Supporting information.

## Data Availability

The data that support the findings of this study are available on request from the corresponding author. The data are not publicly available due to privacy or ethical restrictions.

## References

[1] M. Hughes and C. Duffy , “Public Involvement in Health and Social Sciences Research: A Concept Analysis,” Health Expectations 21, no. 6 (2018): 1183–1190. 30159960 10.1111/hex.12825 PMC6250854

[2] “Official Statistics Liver Disease Profiles ,” Office for National Statistics (2023), https://www.gov.uk/government/statistics/liver-disease-profiles-march-2023-update/liver-disease-profiles-march-2023-update.

[3] B. Kimbell , K. Boyd , M. Kendall , J. Iredale , and S. A. Murray , “Managing Uncertainty in Advanced Liver Disease: A Qualitative, Multiperspective, Serial Interview Study,” BMJ Open 5, no. 11 (2015): e009241. 10.1136/bmjopen-2015-009241 PMC4654301 26586325

[4] J. Low , S. Davis , V. Vickerstaff , et al., “Advanced Chronic Liver Disease in the Last Year of Life: A Mixed Methods Study to Understand How Care in a Specialist Liver Unit Could Be Improved,” BMJ Open 7, no. 8 (2017): e016887. 10.1136/bmjopen-2017-016887 PMC5623344 28851793

[5] T. G. Bird , P. Ramachandran , and E. Thomson , “Decompensated Liver Cirrhosis,” Anaesthesia & Intensive Care Medicine 16, no. 4 (2015): 180–185.

[6] J. K. Peng , N. Hepgul , I. J. Higginson , and W. Gao , “Symptom Prevalence and Quality of Life of Patients With End‐Stage Liver Disease: A Systematic Review and Meta‐Analysis,” Palliative Medicine 33, no. 1 (2019): 24–36. 30345878 10.1177/0269216318807051 PMC6291907

[7] H. Woodland , B. Hudson , K. Forbes , A. McCune , and M. Wright , “Palliative Care in Liver Disease: What Does Good Look Like?” Frontline Gastroenterology 11, no. 3 (2019): 218–227. 32419913 10.1136/flgastro-2019-101180 PMC7223359

[8] H. Woodland , R. M. Buchanan , A. Pring , et al., “Inequity in End‐of‐Life Care for Patients With Chronic Liver Disease in England,” Liver International 43, no. 11 (2023): 2393–2403. 37519025 10.1111/liv.15684

[9] D. Das , M. Ali , I. A. Hussain , et al., “What Do We Know About Patients' Perspectives and Expectations Relating to Palliative and End‐of‐Life Care in Advanced Liver Disease? A Systematic Review of Qualitative Literature Using Entreq Guidelines,” *BMJ Supportive & Palliative Care*. Published ahead of print, 2021. 10.1136/bmjspcare-2021-003057. 34233896

[10] C. J. Beresford , L. Gelling , S. Baron , and L. Thompson , “The Experiences of People With Liver Disease of Palliative and End‐of‐Life Care in the United Kingdom—A Systematic Literature Review and Metasynthesis,” Health Expectations 27, no. 1 (2024): e13893. 10.1111/hex.13893 PMC10768859 37855242

[11] “Briefing Notes for Researchers—Public Involvement in NHS, Health and Social Care Research ,” National Institute for Health Research (NIHR) (2021), https://www.nihr.ac.uk/documents/briefing-notes-for-researchers-public-involvement-in-nhs-health-and-social-care-research/27371#:~:text=Public%20involvement%20in%20research%20is,that%20is%20relevant%20to%20them.

[12] “What is Public Involvement in Research? ” Health Research Authority (2023), https://www.hra.nhs.uk/planning-and-improving-research/best-practice/public-involvement/.

[13] S. Dawson , A. Ruddock , V. Parmar , et al., “Patient and Public Involvement in Doctoral Research: Reflections and Experiences of the PPI Contributors and Researcher,” Research Involvement and Engagement 6, no. 1 (2020): 23. 32426162 10.1186/s40900-020-00201-w PMC7216324

[14] B. Kimbell , “Living, Dying and Caring in Advanced Liver Disease: The Challenge of Uncertainty” (PhD thesis, University of Edinburgh, 2014).

[15] D. Hull , D. Barton , K. Guo , C. Russell , B. Aucott , and D. Wiles , “Patient and Public Involvement to Support Liver Disease Research,” British Journal of Nursing 21, no. 16 (2012): 972–976. 23123652 10.12968/bjon.2012.21.16.972

[16] J. Ocloo , S. Garfield , B. D. Franklin , and S. Dawson , “Exploring the Theory, Barriers and Enablers for Patient and Public Involvement Across Health, Social Care and Patient Safety: A Systematic Review of Reviews,” Health Research Policy and Systems 19, no. 1 (2021): 8. 33472647 10.1186/s12961-020-00644-3 PMC7816359

[17] P. Beresford , It's Our Lives: A Short Theory of Knowledge, Distance and Experience (London, UK: Shaping our Lives in partnership with Citizen Press, 2003).

[18] P. Beresford , “PPI or User Involvement: Taking Stock From a Service User Perspective in the Twenty First Century,” Research Involvement and Engagement 6 (2020): 36. 32601547 10.1186/s40900-020-00211-8 PMC7317269

[19] K. Charmaz , Constructing Grounded Theory. Second edition (London, UK: Sage Publications, 2014).

[20] J. K. Dickinson , D. Bialonczyk , J. Reece , et al., “Person‐First Language in Diabetes and Obesity Scientific Publications,” Diabetic Medicine 40 (2023): e15067. 36786059 10.1111/dme.15067

[21] M. Carol , M. Pérez‐Guasch , E. Solà , et al., “Stigmatization is Common in Patients With Non‐Alcoholic Fatty Liver Disease and Correlates With Quality of Life,” PLoS One 17, no. 4 (2022): e0265153. 35385510 10.1371/journal.pone.0265153 PMC8986095

[22] “Official Statistics Liver Disease Profiles: November 2021 Update ,” Office for Health Improvement and Disparities (2021), https://www.gov.uk/government/statistics/liver-disease-profiles-november-2021-update/liver-disease-profiles-november-2021-update#:~:text=2.-,Main%20findings,areas%20being%20more%20adversely%20affected.

[23] P. Beresford , Beyond the Usual Suspects (London, UK: Shaping our Lives, 2013).

[24] “What is Public Involvement? ,” National Institute for Health Research (NIHR) (2023), https://sphr.nihr.ac.uk/public-involvement/what-is-public-involvement/.

[25] G. Rolfe , D. Freshwater , and M. Jasper , Critical Reflection in Nursing and the Helping Professions: A User's Guide (Basingstoke, UK: Palgrave Macmillan, 2001).

[26] B. Bowness , C. Henderson , S. C. Akhter Khan , M. Akiba , and V. Lawrence , “Participatory Research With Carers: A Systematic Review and Narrative Synthesis,” Health Expectations 27, no. 1 (2024): e13940. 10.1111/hex.13940 PMC10734554 39102730

[27] J. Reynolds and R. Beresford , “‘An Active, Productive Life’: Narratives of, and Through, Participation in Public and Patient Involvement in Health Research,” Qualitative Health Research 30, no. 14 (2020): 2265–2277. 33012242 10.1177/1049732320961053 PMC7649936

[28] S. E. Knowles , D. Allen , A. Donnelly , et al., “More Than a Method: Trusting Relationships, Productive Tensions, and Two‐way Learning as Mechanisms of Authentic Co‐Production,” Research Involvement and Engagement 7, no. 1 (2021): 34. 34059159 10.1186/s40900-021-00262-5 PMC8165763

[29] A. Rahman , S. Nawaz , E. Khan , and S. Islam , “Nothing About Us, Without Us: Is For Us,” Research Involvement and Engagement 8, no. 1 (2022): 39. 35927767 10.1186/s40900-022-00372-8 PMC9351243

[30] “Official Statistics Liver Disease Profiles, January 2022 Update ,” Office for Health Improvement and Disparities (2022), https://www.gov.uk/government/statistics/liver-disease-profiles-january-2022-update/liver-disease-profiles-january-2022-update.

[31] W. Alazawi , R. Mathur , K. Abeysekera , et al., “Ethnicity and the Diagnosis Gap in Liver Disease: A Population‐Based Study,” British Journal of General Practice 64, no. 628 (2014): e694–e702. 10.3399/bjgp14X682273 PMC4220229 25348993

