# Supplementary material for: Embedding Public Involvement in a PhD Research Project With People Affected by Advanced Liver Disease
Source: Health Expect. 2024 Jun 12;27(3):e14097. doi: 10.1111/hex.14097 (PMC11167232; doi:10.1111/hex.14097)
Supplement: Supplementary file 1 — Supporting information. [file HEX-27-e14097-s001.pdf]

Appendix 1. VOICE advert (from voice-global.org)

Voice Browse What is Voice About Ac

Advanced liver disease: experiences of care.

BOURNEMOUTH UNIVERSITY • **Online workshop** • posted 1 year ago

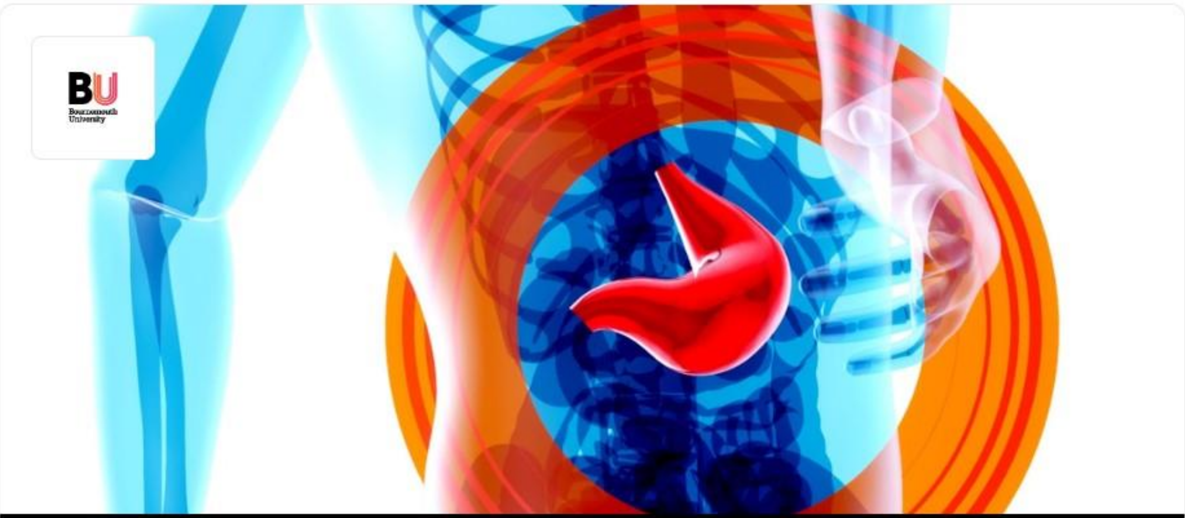

Closes Tue 4th Apr 12:00pm

Date & time
Tuesday 18 April, 2023
11:30 - 13:00

Language
English

Reward
£25 Amazon voucher for involvement in the workshop

Experience
We are looking for 8-10 people to take part. You can get involved if you have liver disease, or if you have experience as a family member/carer of someone with liver disease within the last 2 years.

About

In the UK, liver disease is a growing health concern. Liver disease causes challenging symptoms, emotional and social issues for people affected by it. Increasingly it is understood that people with liver disease, and their carers, should be offered early support to manage these. Our research project aims to explore the experiences of people receiving care for advanced liver disease. We are in the planning stages of the research and are keen for VOICE members to be involved in the study design to ensure that it is meaningful to people with liver disease.
