# Supplementary material for: Embedding Public Involvement in a PhD Research Project With People Affected by Advanced Liver Disease
Source: Health Expect. 2024 Jun 12;27(3):e14097. doi: 10.1111/hex.14097 (PMC11167232; doi:10.1111/hex.14097)
Supplement: Supplementary file 2 — Supporting information. [file HEX-27-e14097-s002.pdf]

Experiences of care in advanced liver disease study

ISSUE 1

Study IRAS Number: 331914

Public involvement newsletter January 2024

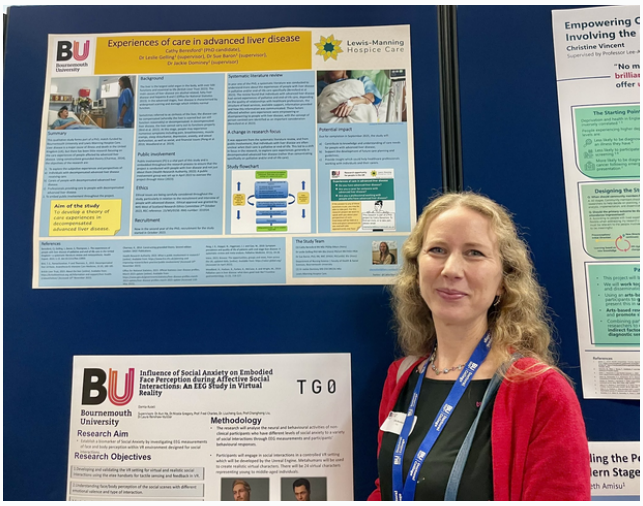

Thank you very much for your involvement in the study. This newsletter is to update you on my progress since the last public involvement meeting. Feel free to get in touch if you have any comments or suggestions about the project, which is match-funded by Bournemouth University and Lewis-Manning Hospice Care.

Lewis-Manning Hospice Care

Happy New Year to you all...

Following on from our last public involvement meeting 30th November, the main focus has been recruitment to the study. It is proving challenging! So far, I have recruited and interviewed:

- One person who was a carer for an individual with advanced liver disease.
- Six healthcare professionals working in liver disease (five are specialist nurses and one is a psychologist). I am holding-off recruiting anymore healthcare professionals now because the priority is speaking with people who have advanced liver disease.
- One person with advanced liver disease.

Save the date:

The next public involvement meeting will be **Wednesday 6th March 10am-11:30am** via Teams. You will receive a £25 Amazon voucher for participation in the meeting, where we will discuss some of the initial findings.

How am I reaching people?

As you suggested at the last meeting, I have been sharing the research far and wide to give people the opportunity to take part in the study. Strategies include:

- Social media: Facebook, X (Twitter) and WhatsApp.
- Liver charities – LIVErNORTH have shared the advert, and I am waiting to find out if the British Liver Trust will help.
- I have shared with specialist liver nurses and doctors for them to inform their patients.
- I have meetings arranged with two hospices, who may be able to help.
- I have a couple of GP contacts who have agreed to share it.
- I have contacted organisations supporting homeless people (because liver disease is more common in individuals experiencing homelessness).

Let me know if you have any other suggestions for recruitment - thank you.

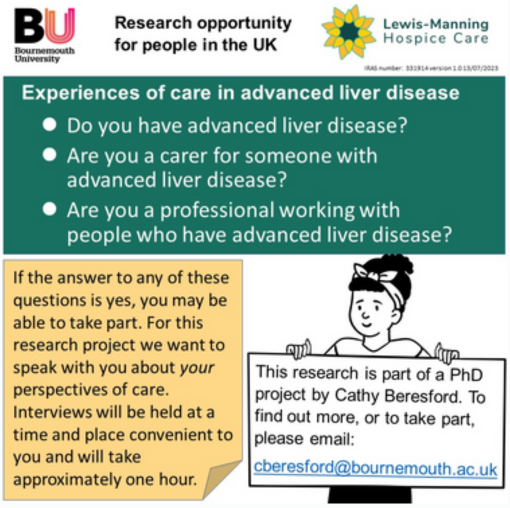

Other activities:

- I presented a poster at Bournemouth University postgraduate conference in November.
- The talk I did for LIVErNORTH is available on YouTube: Catherine Beresford. Experiences of Care in Advanced Liver Disease - YouTube
- I am presenting a poster at Sigma nursing conference at Bournemouth University in June about the project.
- I plan to present at the Faculty of Health and Social Sciences conference in June at Bournemouth University.

Keep in touch: cberesford@bournemouth.ac.uk

For information and advice about your liver, visit: Britishlivertrust.org.uk
